# Impact of high temperature stress on floret fertility and individual grain weight of grain sorghum: sensitive stages and thresholds for temperature and duration

**DOI:** 10.3389/fpls.2015.00820

**Published:** 2015-10-06

**Authors:** P. V. V. Prasad, Maduraimuthu Djanaguiraman, Ramasamy Perumal, Ignacio A. Ciampitti

**Affiliations:** ^1^Department of Agronomy, Throckmorton Plant Science Center, Kansas State UniversityManhattan, KS, USA; ^2^Agricultural Research Center – HaysHays, KS, USA

**Keywords:** abiotic stress, floret fertility, grain size, reproductive success, sensitive stage, spikelet fertility, sporogenesis

## Abstract

Sorghum [*Sorghum bicolor* (L.) Moench] yield formation is severely affected by high temperature stress during reproductive stages. This study pursues to (i) identify the growth stage(s) most sensitive to high temperature stress during reproductive development, (ii) determine threshold temperature and duration of high temperature stress that decreases floret fertility and individual grain weight, and (iii) quantify impact of high daytime temperature during floret development, flowering and grain filling on reproductive traits and grain yield under field conditions. Periods between 10 and 5 d before anthesis; and between 5 d before- and 5 d after-anthesis were most sensitive to high temperatures causing maximum decreases in floret fertility. Mean daily temperatures >25°C quadratically decreased floret fertility (reaching 0% at 37°C) when imposed at the start of panicle emergence. Temperatures ranging from 25 to 37°C quadratically decreased individual grain weight when imposed at the start of grain filling. Both floret fertility and individual grain weights decreased quadratically with increasing duration (0–35 d or 49 d during floret development or grain filling stage, respectively) of high temperature stress. In field conditions, imposition of temperature stress (using heat tents) during floret development or grain filling stage also decreased floret fertility, individual grain weight, and grain weight per panicle.

## Introduction

Grain sorghum [*Sorghum bicolor* (L.) Moench] is an important dryland crop for food, feed, and fuel. In the semi-arid tropics the mean crop growing season temperatures are already close to optimum or above optimum for grain sorghum growth and development (Prasad et al., 2006a; Singh et al., 2014). The optimum range of air temperature for sorghum during the vegetative period is 26–34°C (Hammer et al., 1993); and during the reproductive period is 25–28°C (Prasad et al., 2006a, 2008a). Future projected changes in temperature could severely impact sorghum yields in several parts of the world. Srivastava et al. (2010) projected that climate change (HadCM3 model, A2a scenario) in different parts of India will decrease rainy season sorghum yields up to 14% and post rainy season sorghum yields up to 7% by 2020. Similarly, Butt et al. (2005) projected a decrease in sorghum yield from 11 to 17% in Mali by 2030. Several other simulation models projected decrease in sorghum yield under future climate scenarios in Africa (Chipanshi et al., 2003; Tingem et al., 2008; Sultan et al., 2013, 2014). Projections for middle and end of the century are more severe.

Sorghum yield potential is often limited by short high temperature stress episodes occurring primarily during the reproductive period (Prasad et al., 2008a). Occurrence and intensity of high-temperature episodes is likely to increase in future climates (IPCC, 2013). Increased frequency of high temperature stress can cause significant yield losses depending on timing (sensitive growth stages), intensity and duration. Thus, it is critical to precisely: (i) quantify temperature responses, (ii) determine threshold for absolute temperature values and its duration, and (iii) define sensitive growth stages. Such information is needed to accurately estimate implications of climate variability and understand impact of short episodes of high temperature events on sorghum grain yield and its components.

Grain crops are generally more sensitive to high temperatures during reproductive than vegetative stages of crop development (Farooq et al., 2011); primarily impacting yield formation. The two major yield components of grain crops are grain numbers and individual grain weight (grain size), both of which are sensitive to high temperatures. Grain numbers are a result of successful fertilization (seed-set), which mainly depends on the functionality of male (pollen) and female (ovule) gametes. Adverse environmental conditions during floral development and anthesis can negatively influence viability and functionality of gametes leading to decreases in floret fertility and, consequently, seed set. Similarly, high temperatures during the grain filling period decrease individual grain size due to shorter grain filling duration (Prasad et al., 2008a) and/or grain filling rate (Prasad et al., 2006a, 2008b; Dias and Lidon, 2009). Decreases in grain number and individual grain weight leads to lower grain yields.

High temperature stress during pre-anthesis (sporogenesis) decreases pollen viability and fewer pollen grains, resulting in decreased seed set in grain sorghum (Prasad et al., 2008a). High temperature stress during floret development alters pollen morphology and results in an abnormal exine wall, degeneration of tapetum cells, and membrane damage, leading to pollen sterility in grain sorghum (Djanaguiraman et al., 2014), wheat (Prasad and Djanaguiraman, 2014), and soybean (Djanaguiraman et al., 2013a,b). Similarly, high temperature stress during anthesis decreases seed set in many cereal crops including sorghum (Prasad et al., 2008a; Jain et al., 2010; Prasad and Djanaguiraman, 2011; Nguyen et al., 2013; Djanaguiraman et al., 2014; Singh et al., 2015), wheat (*Triticum aestivum* L.) (Prasad et al., 2008b; Narayanan et al., 2015), rice (*Oryza sativa* L.) (Jagadish et al., 2007, 2010); and legume crops such as peanut (*Arachis hypogaea* L.) (Prasad et al., 1999, 2001), and soybean (*Glycine max* L. Merr.) (Djanaguiraman et al., 2013a,b) resulting in lower grain numbers and grain yield. High temperature stress during anthesis causes poor anther dehiscence and impairs pollen tube growth and hampers fertilization, resulting in lower seed set (Prasad et al., 2006b; Jagadish et al., 2007).

Although studies have shown that in grain sorghum episodes of high temperature stress (>36°C; daytime maximum temperature) decreases floret fertility (Prasad et al., 2008a; Jain et al., 2010), the relative sensitivity of various reproductive growth stages (panicle emergence, anthesis, and grain filling), critical thresholds for temperatures, and duration of stress have not been thoroughly quantified. In addition, the effect of high temperature stress on field grown sorghum is not well documented. The specific objectives of this research were to (i) identify the growth stage(s) most sensitive to high temperature stress during reproductive development, (ii) determine threshold temperature and duration of high temperature stress that decreases floret fertility and individual grain weight, and (iii) quantify impact of high daytime temperature during floret development, flowering and grain filling on reproductive traits and yield components for grain sorghum grown under field conditions.

## Materials and Methods

This research was conducted at facilities established in the Department of Agronomy at Kansas State University, Manhattan, KS, USA. A series of experiments, under controlled environment and field condition, were conducted to quantify impact of high temperature stress on reproductive traits and grain yield of grain sorghum crop.

### Plant Husbandry and Growth Conditions

Photoperiod-insensitive grain sorghum hybrid DK28E (short stature, early maturity, short duration, and stands well under stress conditions; DeKalb company) was used in this study due its sensitivity to high temperature stress (Prasad et al., 2006a, 2008a). Seeds were treated with fungicide (Captan, Hummert International, Earth City, MO, USA) as a precautionary measure against seed-borne diseases and sown at 4-cm depth in 15-L pots (pot diameter at the top and bottom was 27.5 and 26 cm, respectively; pot depth was 22 cm) containing commercial potting soil (Metro Mix 350, Hummert International, Topeka, KS, USA). After emergence, plants were thinned to one plant per pot and maintained until maturity. A systemic insecticide, Marathon 1%G (granules) (a.i. Imidacloprid 1-[(6-Chloro-3-pyridinyl) methyl]-N-nitro-2-imidazolidinimine, Hummert International, KS, USA) was applied to each pot at 4 g per pot. Each pot was fertilized with Osmocote (controlled release plant food, 14:14:14%, N: P_2_O_5_: K_2_O, respectively; Hummert International, KS, USA) at 5 g per pot; applied before sowing and at flowering. To avoid water stress, all pots were irrigated daily from sowing to maturity.

Sorghum plants were grown in four large growth chambers (PGW 36, about 249 cm wide, 137 cm deep, and 180 cm high, Conviron, Winnipeg, MB, Canada) from sowing to start of high temperature stress treatments. Five pots were transferred for implementing various temperature and duration treatments to six other similar but slightly smaller growth chambers (PGW 15, about 184 cm wide, 78 cm deep, and 130 cm tall, Conviron, Winnipeg, MB, Canada). The quality of environmental conditions was similar in all growth chambers. After the temperature treatment, pots were returned back to the original growth chamber where they remained until final harvest at maturity. The pots were randomly arranged within each growth chamber. Plants in each growth chamber were moved randomly every 7–10 d during non-stress and every 1–2 d during the stress periods to avoid positional effects within the chamber. Temperatures in growth chambers were maintained in a square wave fashion. In all temperature regimes, daytime and nighttime temperatures were held for 12 h and the transition period between the daytime maximum and nighttime minimum temperatures was 6 h, and vice versa. Relative humidity (RH) in all growth chambers was set at 80% to avoid any confounding effects of dry air (drought stress). The photoperiod was 12 h (from 0800 to 2000 h). Such temperature conditions often occurs during sensitive stages of crop development in semi-arid, arid and humid regions of US, Africa and Asia (Prasad et al., 2006a, 2008a). In all the growth chambers, the canopy level photon flux density (400–700 nm) was close to 900 μmol m^-2^ s^-1^ provided by cool white fluorescent lamps (Philips Lighting Co., Somerset, NJ, USA). Air temperature and RH were continuously monitored at 15-min intervals in all growth chambers throughout the experiment using HOBO data loggers (Onset Computer Corporation, Bourne, MA, USA).

### Treatments and Observations

#### Impact of High Temperature Stress: Sensitive Stages during Reproductive Development

Grain sorghum plants were grown under optimum temperature (30/20°C, daytime maximum and nighttime minimum temperature; 12 h photoperiod, and 80% humidity) from seedling emergence until onset of panicle emergence (about 15 d before anthesis). Thereafter, a set of five pots was transferred from the optimum temperature to high temperature stress conditions (36/26°C daytime maximum/nighttime minimum, 12 h photoperiod, and 80% humidity) at 5-days intervals, starting from 15 d before anthesis to 30 d after anthesis (a total of 10 treatments). The duration of high temperature stress for each treatment was 5 d. After the stress period, each set of five pots corresponding to a different treatment was returned to optimum temperature, where it remained until final harvest at maturity. Control plants (five pots) remained under optimum temperature from seedling emergence to final harvest at maturity.

In each treatment, florets on the middle portion of the panicle on each plant were tagged with cotton thread. This portion covered about 15–20 branches on each panicle. On each panicle about 80–100 florets were marked with permanent ink marker and used to determine floret fertility. At maturity, the tagged florets were hand-harvested and dried at 40°C for 7 d. Individual marked florets were checked for grain by pressing the floret between the thumb and the index finger. Both partially and fully filled tagged florets were used to determine floret fertility. Floret fertility percentage was estimated as the ratio of the total number of tagged florets to the number of grains from the tagged florets. The tagged florets were hand-threshed, counted, and weighted. Individual grain weight was calculated by dividing the total grain weight by number of grains from the tagged florets. Data on floret fertility and individual grain weight of various high temperature treatments are presented as percentage of control treatment (optimum temperature).

#### Impact of High Temperature Stress: Threshold Temperature during Floret Development and Grain Filling

Threshold temperatures during floret development were evaluated in growth chamber conditions with sorghum plants growing under optimum temperature (30/20°C, daytime maximum/nighttime minimum; 12 h photoperiod, and 80% humidity) from seedling emergence to start of panicle emergence (about 15 d before anthesis). Thereafter, a set of five pots was transferred from optimum temperature to six different temperature treatments (32/22, 36/26, 38/28, 40/30, 42/32, and 45/35°C, daytime maximum/nighttime minimum temperature, giving daily mean temperatures of 27, 31, 33, 35, 37, and 40°C) for a duration of 10 d. After the stress period, each set of five pots corresponding to different treatments was returned to optimum temperature, where they stayed until final harvest at maturity. Control plants (five pots) remained under optimum temperature from seedling emergence until final harvest at maturity. The procedure of tagging and determining of floret fertility and individual grain weight was similar to as detailed above.

Threshold temperatures during grain filling were quantified with sorghum plants grown in growth chambers under optimum temperature from seedling emergence to start of grain filling (7 d after full anthesis in the middle portion of the panicle). Thereafter, the plants were exposed to different temperature treatments as mentioned above (six treatment combinations). Tagging and floret fertility was determined following a similar procedures detailed above.

#### Impact of High Temperature Stress: Threshold Duration during Floret Development and Grain Filling

To determine threshold duration of high temperature stress during floret development and its subsequent effects on floret fertility, sorghum plants were grown under optimum temperature (30/20°C, daytime maximum/nighttime minimum temperature; 12 h photoperiod, and 80% humidity) from seedling emergence to the start of panicle emergence (about 15 d before anthesis). Thereafter, a set of five pots was transferred from optimum temperature to high temperature (36/26°C, daytime maximum/nighttime minimum temperature) for five different duration treatments (7, 14, 21, 28, and 35 d). After the stress period, each set of five pots corresponding to each treatment was returned to optimum temperature, and remained until maturity. Control plants (five pots) remained under optimum temperature from seedling emergence to maturity. Other procedures for tagging and determining of floret fertility and individual grain weight measurements were similar as detailed in the previous section.

To determine threshold duration of high temperature during grain filling, plants were grown under optimum temperature from seedling emergence to start of grain filling (7 d after full anthesis). The temperature treatments, durations of stress, and procedure of tagging and determination, and presentation of floret fertility and individual grain weight were similar to those mentioned above.

#### Impact of High Temperature Stress: Field Studies using Heat Tents during Floret Development and Grain Filling

To determine the impact of high temperature stress under field conditions, plants of grain sorghum hybrid DK28E were planted at North Farm research field station in Manhattan, Kansas. Standard field crop management practices [76 cm spacing between rows and target plant population of 12 plants m^-2^; 10 g N m^-2^ as urea granules (46% N)] were followed for planting the crop. Pre-emergence herbicide application and hand weeding were done when necessary to keep the plots weed free. Fields were flood irrigated as necessary to ensure that there was no water stress throughout the crop growing season until maturity.

To impose high temperature stress during floret development, three heat tents were placed on the field grown sorghum crop at the start of panicle emergence stage and remained until start of grain filling (about 7 d after full anthesis). Similarly, for imposition of high temperature stress during grain filling, three heat tents were placed on the field grown sorghum crop at the start of grain filling (7 d after full anthesis) and remained until maturity. The procedure of tagging and determination of floret fertility and individual grain weight were similar to those mentioned in previous sections. In addition, data on total number of florets, seeds per panicle, and grain weight per panicle were measured from five tagged plants in each tent (randomly selected).

Each heat tent was built on galvanized steel frame work with additional frame (0.6 m) only on the top that can open and close. The steel frame work is covered by clear polyethylene film which transmits 85% of the incoming solar radiation. Each heat tent was 5.4 m wide, 7.2 m long, and 3.0 m high at the apex. There are no artificial heating systems, heat tents are heated via natural solar radiation (greenhouse effect). Temperature increases inside the heat tents are dependent on the intensity of solar radiation and outside ambient temperatures. On a clear sunny day, the temperature inside the heat tent can be up to 10°C warmer than the outside ambient temperature. Each heat tent was equipped with a solar-powered battery to operate the actuators to open and close the additional frame on the top when temperatures were too high (the maximum was set at 45°C) to avoid excessive heating. Each heat tent has a 15 cm clearance on all four sides to allow circulation of air within the heat tent. Air temperature and RH were measured using WatchDog data loggers (1000 Series Micro Station, Spectrum Technologies, Aurora, IL, USA) at 30 min intervals. Similarly, incoming solar radiation was measured using PAR sensors (LightScout Quantum Light Sensor, Spectrum Technologies, Aurora, IL, USA). Volumetric soil water content was measured at 30 cm depth using soil moisture sensors (WaterScout SM 100 moisture sensor, Spectrum Technologies, Aurora, IL, USA). Environmental measurements were measured inside and outside heat tents.

### Data Analyses

Data from all the experiments were statistically analyzed by using PROC GLM in the SAS software (SAS Institute, Cary, NC, USA). The experimental design for each experiment was a randomized complete block. The temperature of each growth chamber was assigned randomly, and plants (pots) were replicated within the chamber. There were five replications (five pots) for all measurements. Standard error was shown as an estimate of variability, and where appropriate means of different variables were separated by LSD at a probability level of 0.05. The response of floret fertility and individual grain weight to different temperatures and durations was tested for linear or quadratic relationships and significance using regression analysis in SAS and the best fit was identified and presented.

## Results

### Quality Control of Growth Chambers

Mean daytime and nighttime temperatures in the optimum temperature and high temperature treatments were ± 0.5°C of the target temperatures, and RH was within ± 10%. Quality of the temperature control and chamber performance was previously published (Pradhan et al., 2012).

### Impact of High Temperature Stress: Sensitive Stages during Reproductive Development

**FIGURE 1:**
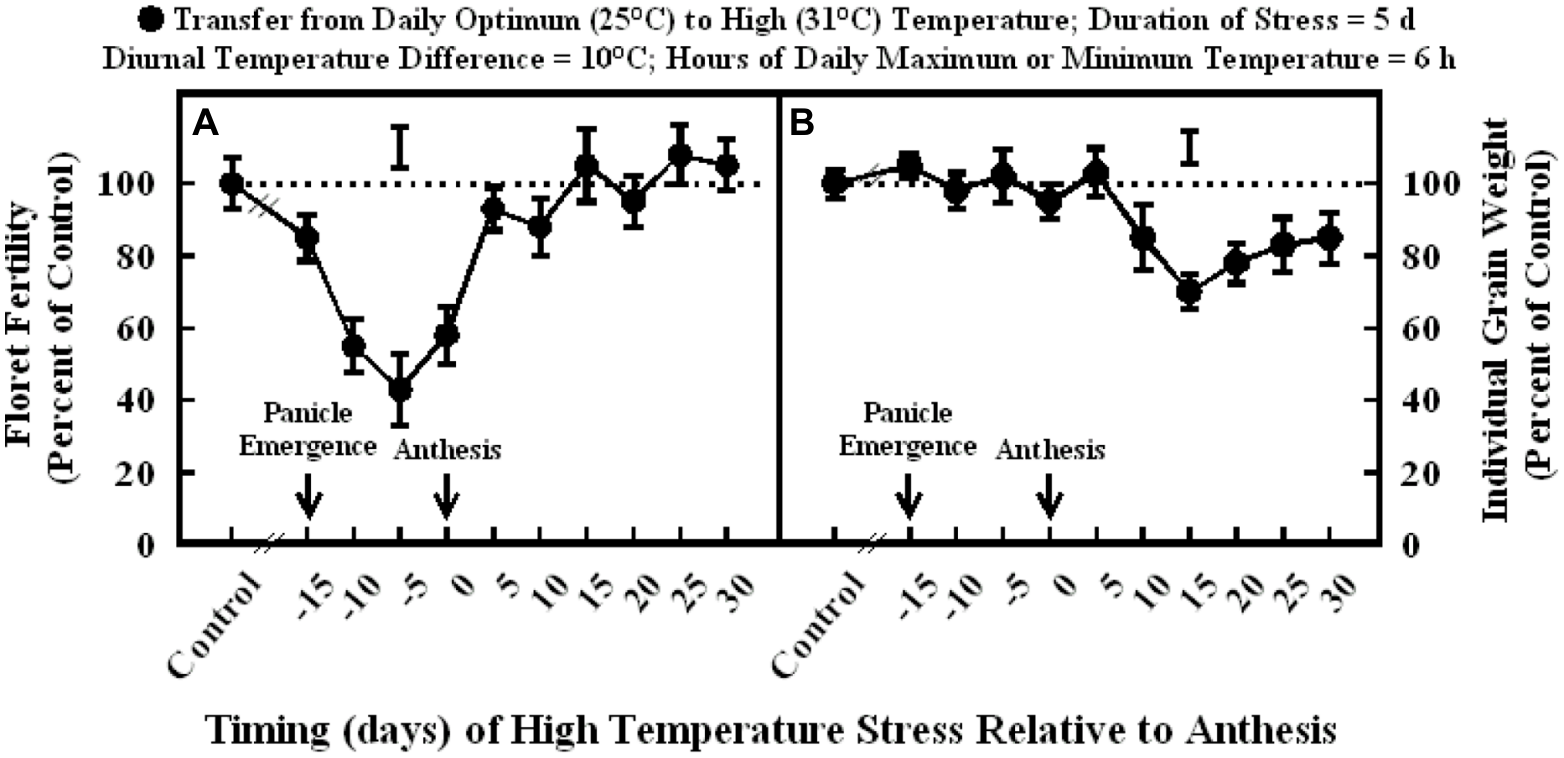
**Impact of high temperature stress (36/26°C daytime maximum/nighttime minimum temperature, for 5 days) at different times relative to anthesis on (A) floret fertility, and (B) individual grain weight.** Each datum is expressed as percentage of control at optimum temperature (30/20°C, daytime maximum/nighttime minimum temperature) and shown with ± SE. Vertical bars above the lines denote LSD for comparison of treatment means. The dotted line provides reference to control means.

Exposure to high temperature stress (36/26°C daytime maximum/nighttime minimum temperature; mean daily temperature, 31°C) for 5 d significantly decreased floret fertility compared to optimum temperature (30/20°C; daytime maximum/nighttime minimum temperature; mean daily temperature, 25°C) when imposed at 15, 10, 5, or 0 d before anthesis and 5 and 10 d after anthesis (**Figure 1A**). Maximum decreases in floret fertility (43–58% of control) occurred when stress was imposed at 10, 5, or 0 d before anthesis. High temperature stress imposed at 15 d before anthesis slightly decreased floret fertility (85% of control) and 5 and 10 d after anthesis (83–92% of control). Heat stress had no influence on floret fertility when stress was imposed at stages occurring beyond 10 d after anthesis (**Figure 1A**).

Exposure to high temperature stress from 15 d before- to 5 d after-anthesis did not influence individual grain weight (**Figure 1B**). However, high temperature stress occurring from 10 to 30 d after anthesis significantly decreased individual grain weight to a similar extent (78–85% of control), except at 15 d after anthesis, which has the maximum decrease (70% of control).

### Impact of High Temperature Stress: Threshold Temperature during Floret Development and Grain Filling

**FIGURE 2:**
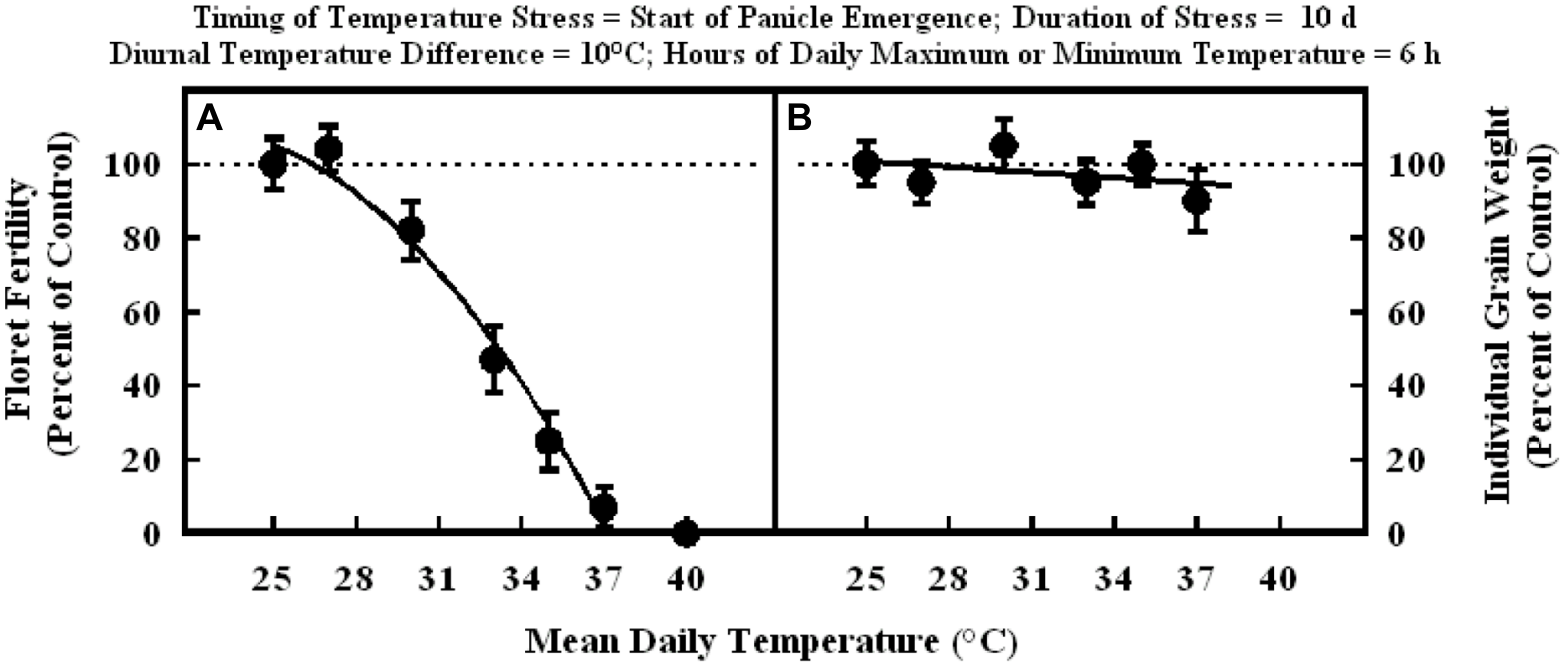
**Impact of different mean daily temperatures (°C) when imposed at start of panicle emergence for a duration of 10 d on (A) floret fertility, fitted line *Y* = –119.7 +20.8X –0.47X^2^; *r*^2^ = 0.98 (*P* < 0.001), and (B) individual grain weight, fitted line *Y* = +112.5 –0.48X; *r*^2^ = 0.18 (NS).** Each datum is expressed as percentage of control at optimum temperature (30/20°C, daytime maximum/nighttime minimum) and shown with ± SE.

Floret fertility decreased significantly with increasing mean daily temperatures in the range of 25–40°C when imposed for a duration of 10 d at the start of panicle emergence. Floret fertility response to temperature fit a quadratic model (**Figure 2A**) Floret fertility decreased from about 100% of control at 25°C mean daily temperature to 0% of control at 37.4°C. Mean daily temperature in the specific range (25 through 37°C) imposed at the start of panicle emergence did not decrease individual grain weight (**Figure 2B**).

**FIGURE 3:**
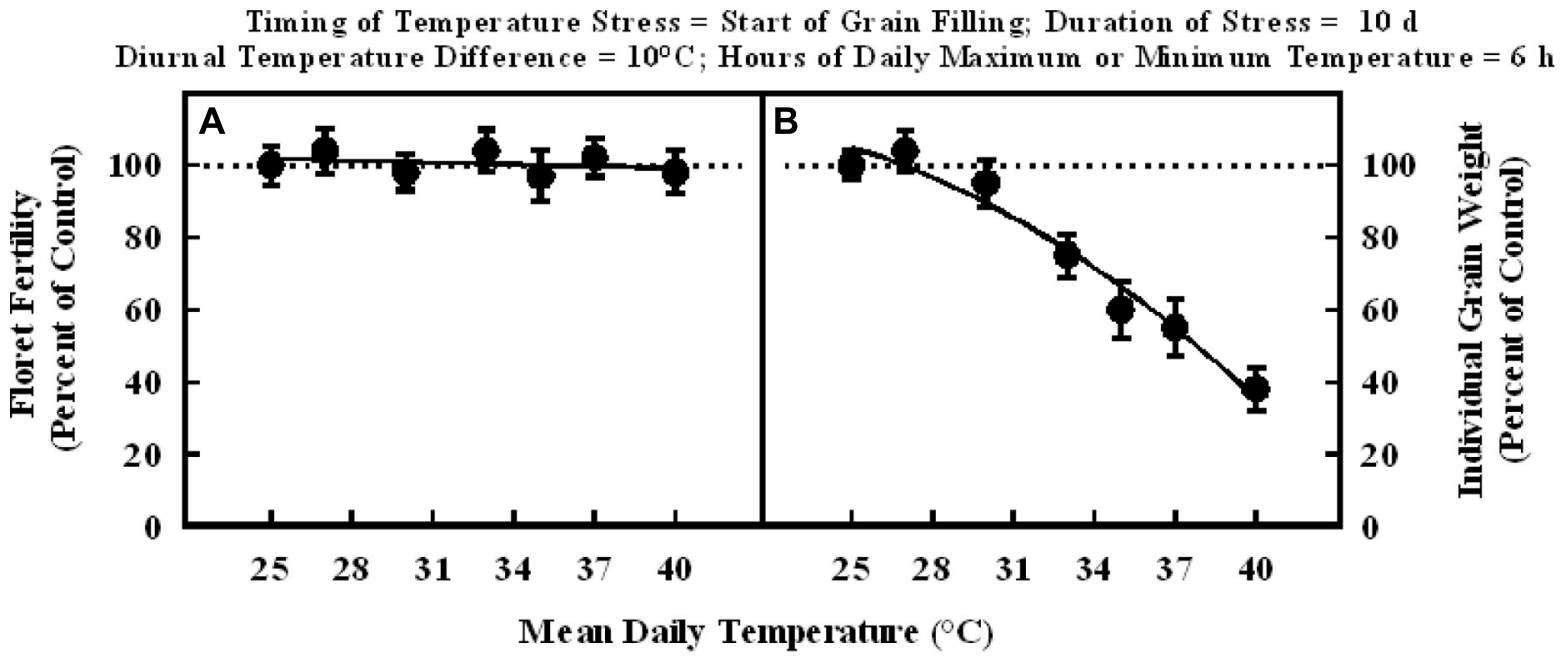
**Impact of different mean daily temperatures (°C) at the start of grain filling for a duration of 10 d on (A) floret fertility, fitted line *Y* = +105.6 – 0.16X; *r*^2^ = 0.09 (NS), and (B) individual grain weight, fitted line *Y* = +66.3 +5.38X –0.15X^2^; *r*^2^ = 0.97 (*P* < 0.001).** Each datum is expressed as percentage of control at optimum temperature (30/20°C, daytime maximum/nighttime minimum) and shown with ± SE.

In the experiment where high temperature stress was imposed at the start of grain filling, there were no significant differences in the floret fertility in all plants and it was within the range of control (**Figure 3A**). Whereas, individual grain weight decreased significantly with increasing mean temperatures in the range of 25–40°C, when imposed for a duration of 10 d at the start of grain filling stage (**Figure 3B**). The grain weight response to temperature was also described with a quadratic model. Individual grain weight decreased by 62% from 25 to 40°C mean daily temperatures (100% of control to about 38% of control at 40°C).

### Impact of High Temperature Stress: Threshold Duration during Floret Development and Grain Filling

**FIGURE 4:**
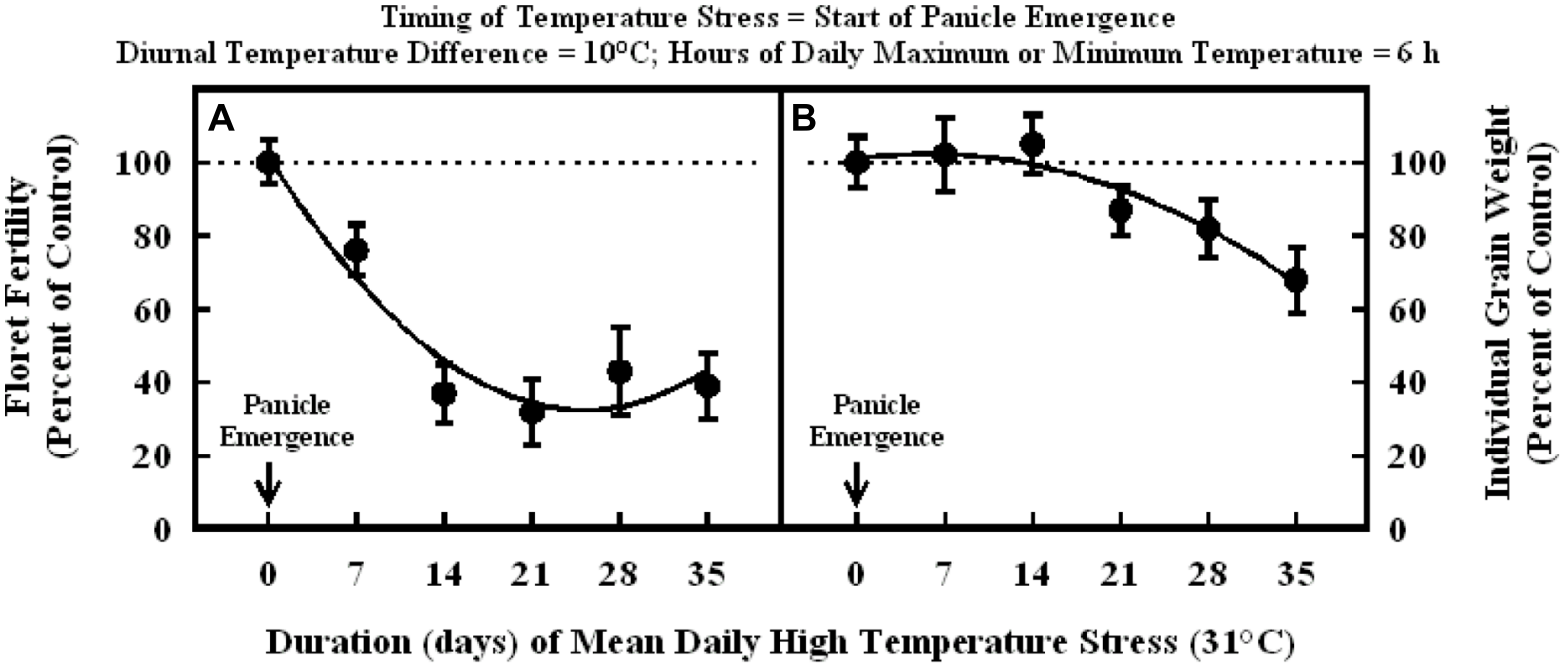
**Impact of high temperature stress (36/26°C, daytime maximum/nighttime minimum) for different durations when imposed at the start of panicle emergence on (A) floret fertility, fitted line *Y* = +101.6 –5.4X +0.11X^2^; *r*^2^ = 0.93 (*P* < 0.001), and (B) individual grain weight, fitted line *Y* = +101 +0.45X –0.041X^2^; *r*^2^ = 0.94 (*P* < 0.001).** Each datum is expressed as percentage of control at optimum temperature (30/20°C, daytime maximum/nighttime minimum) and shown with ± SE.

Plants exposure to high temperature stress (36/26°C; daytime maximum/nighttime minimum temperature; mean daily temperature of 31°C) at the start of panicle emergence caused significant decreases in floret fertility (**Figure 4A**). The response of floret fertility to duration was best described with a quadratic function across all data points (**Figure 4A**). Exposure to high temperature stress for 7 and 14 d periods decreased floret fertility to 76 and 37% of control, respectively. Thereafter, extended duration of the heat stress to 21 and 35 d did not further decrease floret fertility, which remained in the range of 32 and 43% of the control. Individual grain weight was similar between 0, 7, and 14 d of high temperature stress, but further increase in durations to 21, 28, and 35 d decreased individual grain weight to 87, 82, and 68% of control, respectively (**Figure 4B**).

**FIGURE 5:**
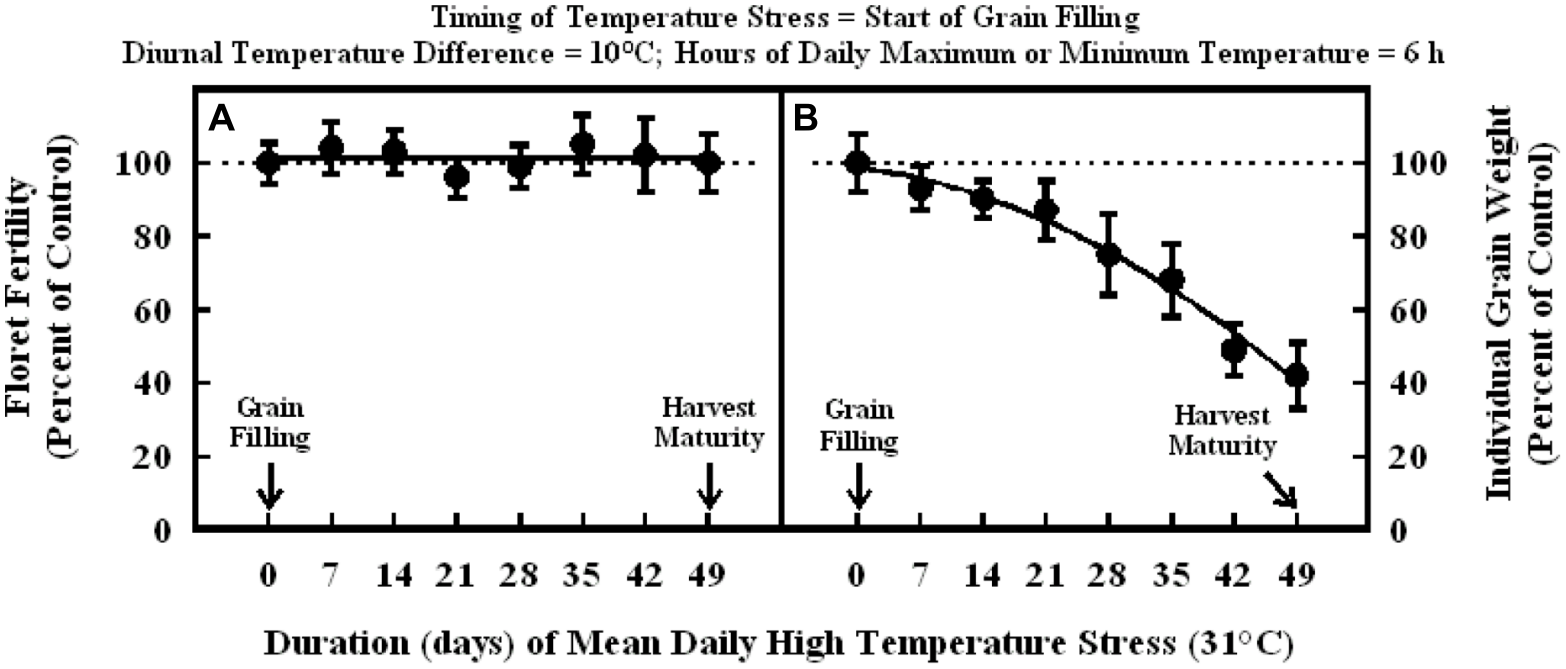
**Impact of high temperature stress (36/26°C, daytime maximum/nighttime minimum) for different durations at the start of grain filling on (A) floret fertility, fitted line *Y* = +101 –0.0017X; *r*^2^ = 0.001 (NS), and (B) individual grain weight, fitted line *Y* = +98.7 – 0.32X –0.018X^2^; *r*^2^ = 0.98 (*P* < 0.001).** Each datum is expressed as percentage of control at optimum temperature (30/20°C, daytime maximum/nighttime minimum) and shown with ± SE.

In the experiment where high temperature stress was imposed at the start of grain filling, there were no significant differences in the floret fertility in all plants and it was within the range of control (**Figure 5A**). Individual grain weight decreased significantly in a quadratic fashion with increasing duration of high temperature stress from 7 to 49 d (**Figure 5B**). Individual grain weights after 7, 14, and 21 d of high temperature stress were in the range of 87 to 93% of control, but when high temperature stress extended to 28 and 35 d, individual grain weight decreased to 75 and 68% of control, respectively. Further increase in the duration of high temperature stress to 42 and 49 d did promote a decrease on individual grain weight to 49 and 42% of control, respectively.

### Impact of High Temperature Stress: Field Studies using Heat Tents during Floret Development and Grain Filling

**FIGURE 6:**
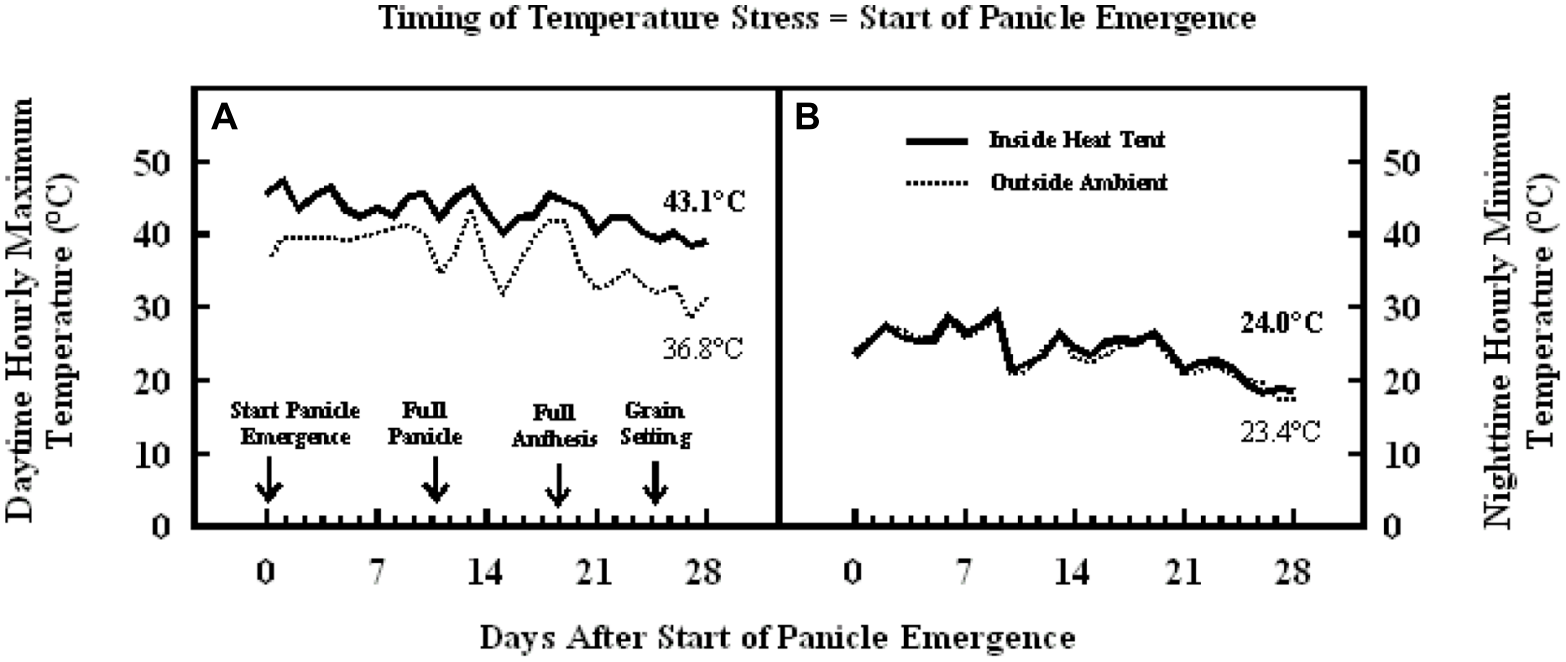
**Data on (A) daytime hourly maximum temperature; and (B) nighttime hourly minimum temperature outside heat tents (ambient, dotted lines) and inside heat tent (high temperature stress, solid lines) from start of panicle emergence to start of grain filling period on field grown sorghum plants during 2011 in Manhattan, KS, USA**.

**FIGURE 7:**
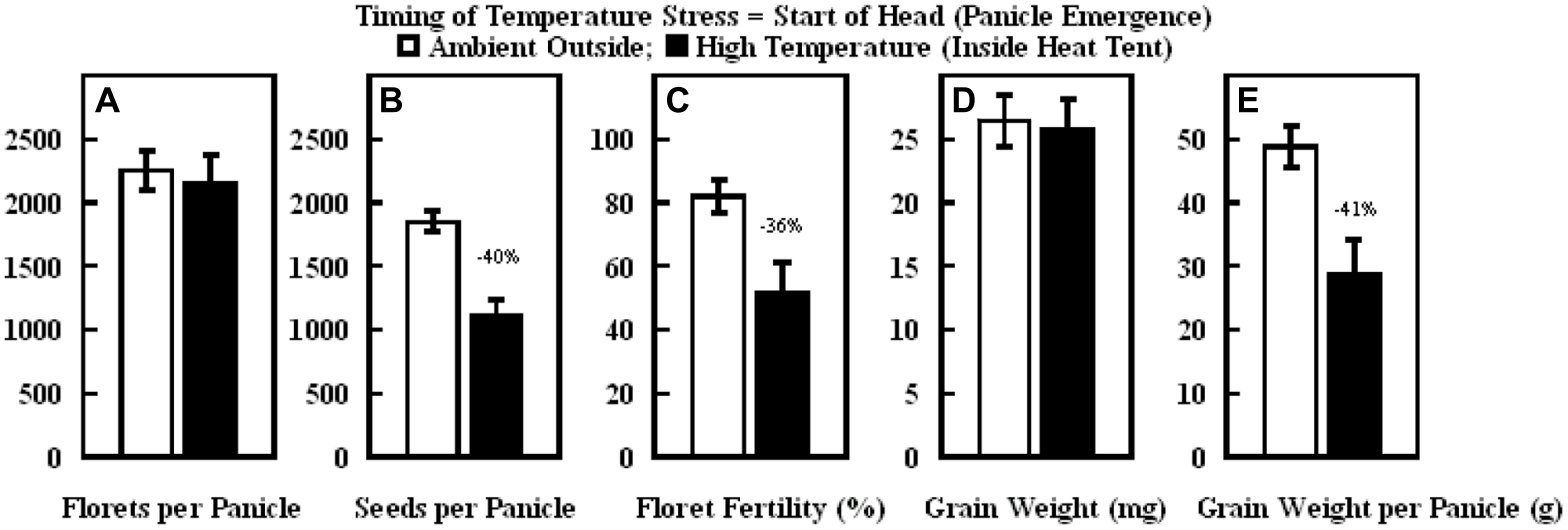
**Impact of high temperature stress imposed by heat tents (43/24°C, daytime maximum/nighttime minimum temperature) relative outside ambient conditions (37/23°C, daytime maximum/nighttime minimum temperature) from start of panicle emergence to start of flowering on (A) number of florets per panicle, (B) number of seeds per panicle, (C) floret fertility (%), (D) individual grain weight (mg), and (E) grain weight per panicle (g).** Each datum is shown with ± SE. Numbers on top of the bars shows the percentage decline under high temperature stress conditions (inside heat tents) from outside ambient conditions.

The daytime hourly mean maximum temperatures from the start of panicle emergence to the start of grain filling outside the heat tent (ambient) was 37°C, while inside the heat tent (high temperature) was 43°C (**Figure 6A**). However, the nighttime hourly mean minimum temperatures outside (ambient) and inside the heat tent were comparable (23–24°C) (**Figure 6B**). These daytime high temperatures above ambient did not cause any difference in number of florets per panicle and individual grain weight, but significantly decreased floret fertility (**Figure 7C**), seeds per panicle (**Figure 7B**), and grain weight panicle (**Figure 7E**) by 36, 40, and 41%, respectively.

**FIGURE 8:**
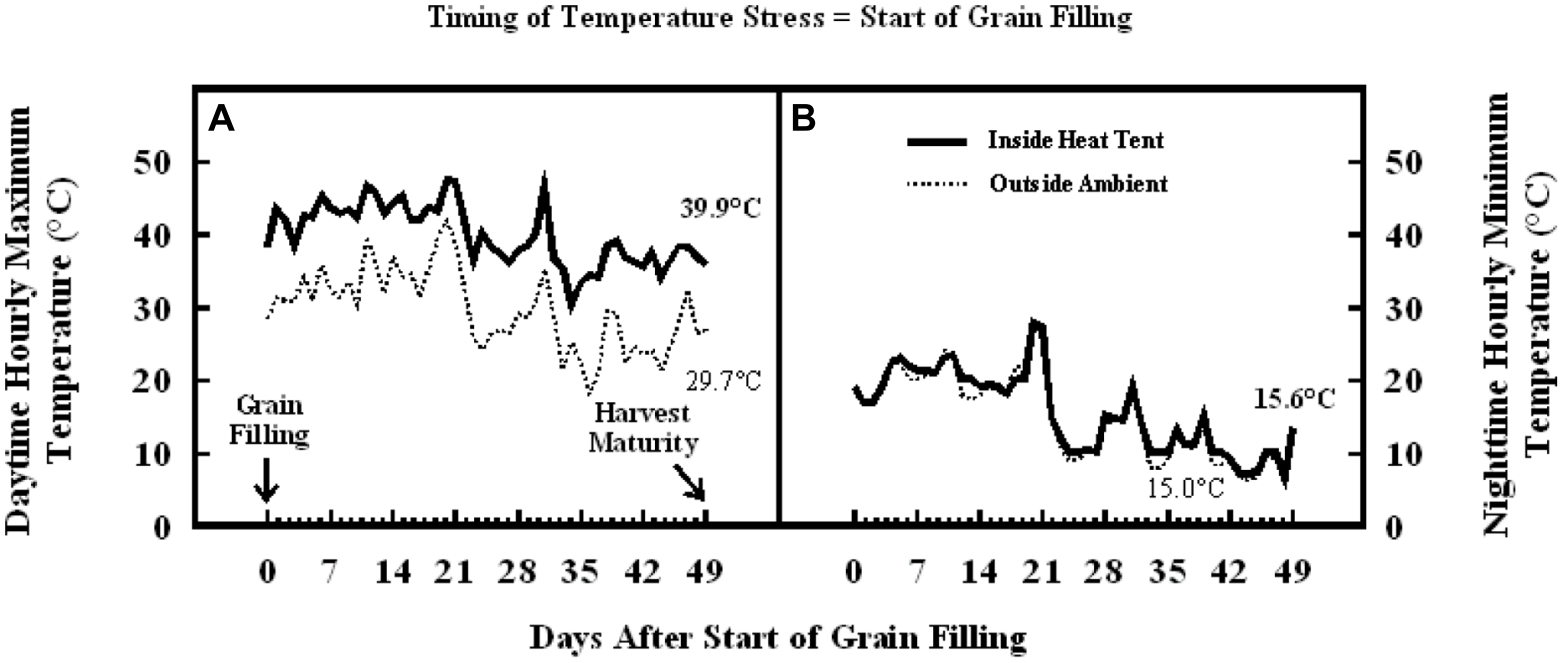
**Data on (A) daytime hourly maximum temperature; and (B) nighttime hourly minimum temperature outside heat tents (ambient, dotted lines) and inside heat tent (high temperature stress, solid lines) from start of grain filling to harvest maturity on field grown sorghum plants during 2011 in Manhattan, KS, USA**.

**FIGURE 9:**
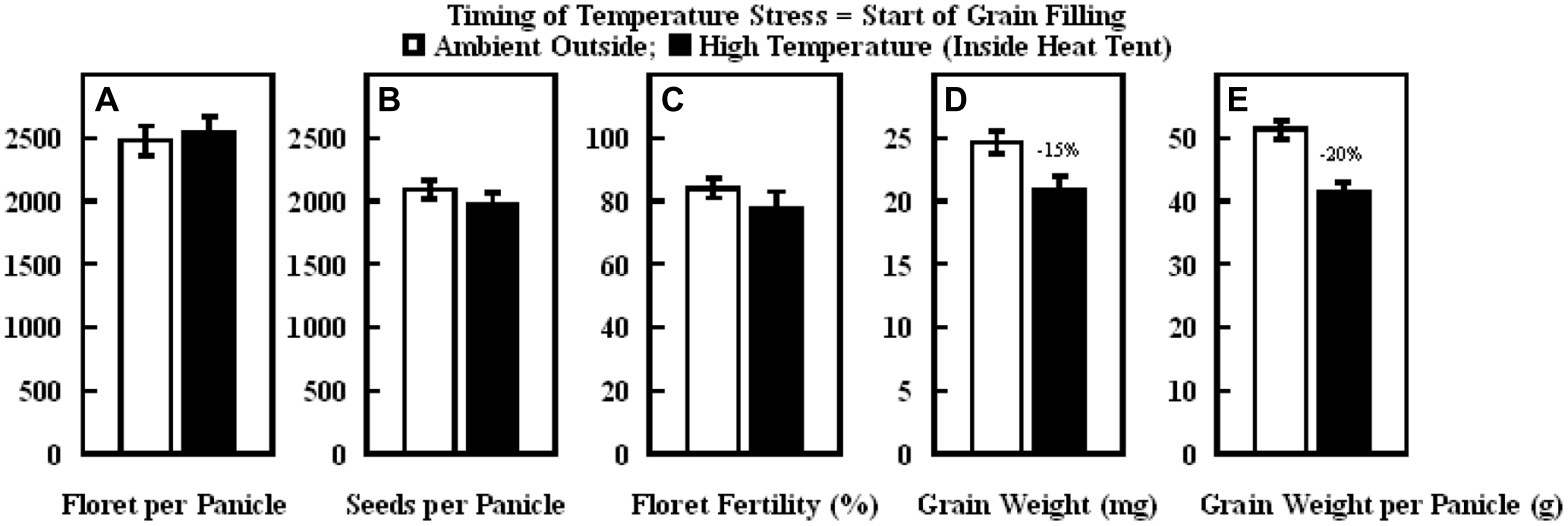
**Impact of high temperature stress imposed by heat tents (40/16°C, daytime maximum/nighttime minimum) relative outside heat tent ambient conditions (30/15°C, daytime maximum/nighttime minimum) from start of grain filling to harvest maturity on (A) number of florets per panicle, (B) number of seeds per panicle, (C) floret fertility (%), (D) individual grain weight (mg), and (E) grain weight per panicle (g).** Each datum is shown with ± SE. Numbers on top of the bars shows the percentage decline under high temperature stress conditions (inside heat tents) from outside ambient conditions.

Similarly, in the experiment where high temperature stress was imposed during grain filling, daytime hourly mean maximum temperature outside the heat tent (ambient) was 30°C, while inside heat tent (high temperature) was 40°C (**Figure 8A**). However, the nighttime hourly mean minimum temperature outside (ambient) and inside heat tent were comparable (15–16°C) (**Figure 8B**). The daytime high temperature stress did not cause any difference in the number of florets per panicle, seeds per panicle or floret fertility, but significantly decreased individual grain weight by 15% and grain weight per panicle by 20% as compared with outside ambient temperatures (**Figure 9**).

## Discussion

In grain sorghum, the most sensitive periods (maximum decreases in floret fertility) to high temperature stress were between 10 and 5 d before anthesis (**Figure 1A**), which coincides with meiosis and tetrad formation stage of sporogenesis, and between 5 d before anthesis and 5 d after anthesis which coincides with anthesis, fertilization, and embryo formation. These periods are similar to those identified in a previous study where the duration of stress was 10 d (Prasad et al., 2008a). A recent study in wheat also showed that the two periods (first at 8–6 d before anthesis and second at 2–0 d before anthesis) during reproductive development were most sensitive to short episodes (2 or 5 d) of high temperature stress, causing maximum decreases in floret fertility (Prasad and Djanaguiraman, 2014). The temperature-sensitive period for cowpea (*Vigna unguiculata* L. Walp.) was from 7 to 9 d before anthesis (Ahmed et al., 1992), for common bean (*Phaseolus vulgaris* L.) from 10 to 12 d before anthesis (Gross and Kigel, 1994), and for peanut about 4 d before anthesis (Prasad et al., 2001).

The loss of floret fertility under high temperature stress during sporogenesis is a result of loss of pollen or ovule viability and/or stigma receptivity (Prasad et al., 2008a; Nguyen et al., 2013; Djanaguiraman et al., 2014; Prasad and Djanaguiraman, 2014). High temperature stress during floret development (sporogenesis) results in several abnormalities in reproductive structures such as the formation of abnormal exines with deeply pitted and non-smooth surface regions and shriveled pollen grains; and desiccated stigma, style, and ovaries (Prasad and Djanaguiraman, 2014). Exine originates from the tapetal cells, and the altered exine ornamentation under high temperature stress is an indication of disruption to tapetal cells. Tapetal cells provide nourishment to the developing pollen, and early degeneration of tapetal cells under high temperature stress affects translocation of nutrients to the developing pollen grains, leading to loss of pollen viability (Hess and Hesse, 1994). These structural and functional abnormalities in reproductive organs lead to floret sterility and failure of fertilization and seed-set leading to lower grain numbers. In addition, studies on grain sorghum showed that high temperature stress causes changes in composition and concentrations of carbohydrates (Jain et al., 2007), structural abnormalities and oxidative damage in pollen gains resulting in pollen sterility and decreased seed-set (Djanaguiraman et al., 2014).

After completion of sporogenesis, the processes of anthesis, fertilization, and embryo formation are also sensitive to high temperature stress. The main processes occurring during this period include dehiscence of anthers, pollination, pollen reception by stigma, pollen germination, pollen tube growth in the style, fertilization, and embryo formation. High temperature stress at the time of anthesis (0 days prior to anthesis or after complete panicle emergence) can decrease floret fertility even when the pollen is viable (Prasad and Djanaguiraman, 2014). The decreased floret fertility under such conditions is commonly due to poor receptivity and dryness of the stigma, poor pollen germination and decreased rate of pollen tube growth, leading to unsuccessful fertilization and lower seed-set (Kakani et al., 2002; Prasad and Djanaguiraman, 2011; Djanaguiraman et al., 2014).

When high temperature stress was imposed for periods of 5 to 14 d during panicle emergence (sporogenesis and anthesis), despite resulting in lower floret fertility (lower grain number) it did not cause any significant increase in subsequent individual grain weights which remained unchanged relative to control plants at optimum temperature either under growth chamber conditions (**Figures 1B**, **2B**, and **4B**) or field conditions under heat tents (**Figure 7**). This shows lack of compensation for fewer grain numbers with increased grain size in this sorghum hybrid. Furthermore, it also suggests that these short periods of high temperature stress during panicle emergence did not trigger subsequent increases in either grain filling rate or grain filling duration, leading to similar grain size. However, when high temperature stress was imposed after embryo formation (with similar seed set and grain numbers), at the start of the grain filling period, there was a quadratic decrease in individual grain weights with increasing temperature stress (**Figure 3B**) or duration of stress (**Figure 5B**). As the duration of high temperature stress increased, individual grain weight decreased in a quadratic fashion (**Figure 4B**). Similar observations were also made when high temperature stress was imposed during grain filling period under field conditions using heat tents (**Figure 9**). This is mainly because final individual grain weight is determined by the rate and duration of grain filling. Decreases in individual grain weight occurs when decreases in grain filling duration is not compensated by increases in grain filling rate. In the research presented here, the responses in grain numbers and grain size to high temperature stress followed a similar trend either in controlled environment growth chambers or field conditions (**Figures 1–3**, **7** and **9**). Our prior research on sorghum showed that season-long high temperature stress did not have a large impact on the rate of grain filling, but it significantly decreased the grain filling duration, leading to smaller individual grain weights (Prasad et al., 2006a). Recent studies on sorghum response to short episodes of high temperature stress under controlled environments and field conditions also showed decreased seed-set and seed numbers; and the response of seed set to high temperature in the field study was well correlated to that of controlled environments (Singh et al., 2015). Furthermore, decreased seed set and seed numbers were not compensated by increased seed size in either controlled environment or field conditions (Singh et al., 2015). There was genetic variability in response to short episodes of high temperature stress in grain sorghum (Nguyen et al., 2013; Singh et al., 2015). Similar results were also observed in wheat (Prasad et al., 2008b). While, decreases in both seed filling rate and seed filling duration were observed in peanut (Prasad et al., 2003).

The results presented on the sensitive stages and thresholds for temperature and duration are from the plants grown in pots and high temperature stress was imposed in growth chambers for fixed duration and following a 10°C diurnal difference between daytime maximum and nighttime minimum temperatures. This was done to ensure full control of temperature regime and avoid confounding effect of other environmental factors. We acknowledge that under field conditions the duration, intensity of temperature stress, daily fluctuations, and diurnal differences of the temperature stress may be highly variable (low or high), but could often be more acute. Although the sensitive stages or periods to high temperature stress may not be different under field conditions, they may have different thresholds. The results of the field studies, following field natural diurnal variations (in the semi-arid environment of Manhattan, Kansas), also showed that high temperature stress induced using heat tents from start of panicle emergence through anthesis decreased floret fertility and consequently grain number, leading to a significant reduction in grain yield (**Figures 7B,C**). Whereas, when high temperature stress was induced during grain filling it resulted in decreased individual grain weight to lower grain yield (**Figures 9D,E**). There were larger diurnal differences under field conditions in heat tents, as heat tents could only increase daytime temperature, while night temperatures were similar (**Figures 6B and 8B**). The results of these field studies complemented growth chambers results presented here and our previous research findings with decreased floret fertility, seed-set percentage, grain numbers and individual grain weight under high temperature stress (Jain et al., 2007, 2010; Prasad et al., 2008a; Nguyen et al., 2013; Djanaguiraman et al., 2014). Studies have shown that increase in nighttime temperatures can cause decreases in sorghum floret fertility, individual grain size, and grain yield (Prasad and Djanaguiraman, 2011). Comparison of daytime and nighttime temperature stress on wheat showed similar decreases in yield and its components (Narayanan et al., 2015). However, more targeted studies to compare influence of day versus night temperatures using appropriate diurnal variations need further investigations. The results presented here are on single genotype, and research has shown that genotypes are known to respond differently to high temperature stress (Nguyen et al., 2013; Djanaguiraman et al., 2014; Singh et al., 2015). Identified sensitive stages will remain the same, but impact on floret fertility and grain weight may change in tolerant versus susceptible sorghum hybrids. There is limited tolerance to high temperature stress in germplasm that is currently being used in breeding programs and there is urgent need for exploiting genotypic resources and systematically evaluating parental lines for tolerance. In addition, there is a critical need for development of high throughput phenotyping techniques to screen large number of germplasm collection and develop biochemical or genetic markers to facilitate efficient breeding for high temperature tolerance.

## Summary and Conclusion

In summary, our research showed that the periods between 10 and 5 d before anthesis (coinciding with sporogenesis) and 5 d before to 5 d after anthesis (coinciding with anthesis, fertilization and embryo formation) were the most sensitive to short episodes (5 d) of high temperature stress (mean daily temperature of 31°C), causing maximum decreases in floret fertility. Mid-duration episodes (10 d) of mean daily temperatures >25°C quadratically decreased floret fertility with the values reaching 0% at 37°C when imposed at start of panicle emergence. Similar increases in mean daily temperatures also quadratically decreased individual grain weight when imposed at start of grain filling. Both floret fertility and individual grain weights decreased quadratically with increasing duration (in the range of 0 to 35 d) of high temperature stress (mean daily temperature of 31°C) when imposed at the start of panicle emergence. Increases in duration (in the range of 0–49 d) of high temperature stress when imposed at the start of grain filling also quadratically decreased individual grain weight. A complementary field study where high daytime temperature stress was imposed using heat tents during floret development decreased floret fertility, seeds per panicle, and grain weight per panicle. Whereas, high daytime temperature stress imposed during grain filling decreased individual grain weight and grain weight per panicle. Further research is underway and required to identify potential parental lines with tolerance to high temperature stress during pre- and post-flowering stages of crop development. In addition, we are focused on determining biochemical or genetic marker(s) that can be used for high throughput phenotyping and facilitate identification of genotypes or parental lines that can be used in breeding for high temperature tolerance. Future research should focus on searching for genotypic variability to be used in breeding programs for building resilience and adaptation to climate change.

## Author Contributions

PP and MD conceived, designed, carried out the experiments and drafted the manuscript. RP and IC made substantial contributions to measurements, data interpretation and edited the manuscript.

## Conflict of Interest Statement

The authors declare that the research was conducted in the absence of any commercial or financial relationships that could be construed as a potential conflict of interest.
